# Glucose challenge increases circulating progenitor cells in Asian Indian male subjects with normal glucose tolerance which is compromised in subjects with pre-diabetes: A pilot study

**DOI:** 10.1186/1472-6823-11-2

**Published:** 2011-01-11

**Authors:** Abel A Nathan, Viswanathan Mohan, Subash S Babu, Soumi Bairagi, Madhulika Dixit

**Affiliations:** 1 Laboratory of Vascular Biology, Department of Biotechnology, Indian Institute of Technology Madras (IIT Madras), Chennai, India; 2 Department of Diabetology, Madras Diabetes Research Foundation (MDRF) & Dr. Mohan's Diabetes Specialities Centre: WHO Collaborating Centre for Non-Communicable Diseases Prevention and Control and IDF Centre for Education, Gopalapuram, Chennai, India; 3 National Institutes of Health-International Center for Excellence in Research, Chennai, India and SAIC Frederick, Inc., NCI Frederick, Frederick, Maryland, USA

## Abstract

**Background:**

Haematopoietic stem cells undergo mobilization from bone marrow to blood in response to physiological stimuli such as ischemia and tissue injury. The aim of study was to determine the kinetics of circulating CD34^+ ^and CD133^+^CD34^+ ^progenitor cells in response to 75 g glucose load in subjects with normal and impaired glucose metabolism.

**Methods:**

Asian Indian male subjects (n = 50) with no prior history of glucose imbalance were subjected to 2 hour oral glucose tolerance test (OGTT). 24 subjects had normal glucose tolerance (NGT), 17 subjects had impaired glucose tolerance (IGT) and 9 had impaired fasting glucose (IFG). The IGT and IFG subjects were grouped together as pre-diabetes group (n = 26). Progenitor cell counts in peripheral circulation at fasting and 2 hour post glucose challenge were measured using direct two-color flow cytometry.

**Results:**

The pre-diabetes group was more insulin resistant (p < 0.0001) as measured by homeostasis assessment model (HOMA-IR) compared to NGT group. A 2.5-fold increase in CD34^+ ^cells (p = 0.003) and CD133^+^CD34^+ ^(p = 0.019) cells was seen 2 hours post glucose challenge in the NGT group. This increase for both the cell types was attenuated in subjects with IGT. CD34^+ ^cell counts in response to glucose challenge inversely correlated with neutrophil counts (ρ = -0.330, p = 0.019), while post load counts of CD133^+^CD34^+ ^cells inversely correlated with serum creatinine (ρ = -0.312, p = 0.023).

**Conclusion:**

There is a 2.5-fold increase in the circulating levels of haematopoietic stem cells in response to glucose challenge in healthy Asian Indian male subjects which is attenuated in subjects with pre-diabetes.

## Background

Haematopoietic stem cell (HSCs) characterized by surface expression of CD34, or CD133 or both exhibit extreme plasticity. Upon receipt of physiological stimuli these cells mobilize from bone marrow into peripheral circulation to mediate multi-lineage engraftment of organs of even non-haematopoietic origin such as skeletal muscle, central nervous system, heart and liver [1]. This pool of immature stem cells is capable of giving rise to progenitors of the cardiovascular system such as the endothelial progenitor cells (EPCs), cardiomyocytes and smooth muscle cell progenitors [2]. They follow circadian kinetics [3] and get mobilized in response to ischemia imparted by acute myocardial infarction (MI) [4]. Similarly during menstrual cycle EPCs follow rhythmic oscillations with maximal increase during ovulatory stage [5].

Abnormalities in carbohydrate metabolism form a continuum which progressively worsens as a subject transitions from NGT to IGT and from IGT to diabetes. This progressive decline in carbohydrate metabolism parallels a concomitant decrease in vascular health and circulating bone marrow-derived vascular progenitor cells [6-11]. Both type 1- and type 2- diabetes are associated with significant reduction in circulating levels of CD34^+ ^cells and EPCs [7,9-13]. Similarly a reduction in the circulating levels of EPCs is seen in IGT subjects [9,14]. However all these studies have looked at progenitor cell counts following overnight fasting and till date no information is available on kinetics of progenitor cells in response to glucose load. Thus the aims of this study were to determine 1) whether levels of CD34^+^, CD133^+ ^and CD133^+^CD34^+ ^progenitor cells increase in circulation in response to glucose load and if it does then 2) is the increase in progenitor counts altered in pre-diabetes subjects exhibiting early stages of compromised carbohydrate metabolism. The interest to better define the relationship between glucose load and progenitor kinetics, also stems from the observation that post-prandial glucose peaks are strongly correlated with increased cardiovascular risks [15,16].

## Methods

### Study Subjects

The study was approved by IIT Madras Institutional ethics committee in accordance with Declaration of Helsinki as revised in 2000 and as required by the Indian Council of Medical Research. Asian Indian male subjects undergoing their first metabolic examination with no prior history of glucose imbalance were recruited at Dr. Mohan's Diabetes Specialties Centre, a tertiary diabetes centre in Chennai, India. The following exclusion criteria were applied: diabetes, established CAD or CVD, smoking, acute infections or immunological disorders, cancer and recent surgery in the past 10 months. Following over-night fast, blood was drawn for quantification of circulating progenitor cells and for determination of their lipid, glucose, insulin and protein profiles. This was followed by 75 g OGTT test and blood was redrawn at 2 hours post glucose load for measurement of progenitor cells, glucose and insulin profiles.

### Definitions and diagnostic criteria

NGT were defined as fasting plasma glucose (FPG) <5.5 mmol/l and 2 hour post load glucose <7.8 mmol/l as per WHO criteria [17]. Pre-diabetes group consisted of subjects with IGT, IFG and combined IGT/IFG. IGT was defined as 2 hour post-load plasma glucose ≥7.8 mmol/l and <11.1 mmol/l as per WHO criteria. IFG was defined based on ADA criteria [17] as FPG ≥5.5 mmol/l and <7 mmol/l and using WHO criteria as FPG ≥ 6.1 mmol/l and <7 mmol/l. Among the total subjects recruited 24 were NGT, 17 were IGT and 9 subjects were IFG.

### Anthropometric measurements

For all subjects the following parameters were recorded: age, body mass index (BMI), height, weight, waist to hip ratio (WHR) and blood pressure. BMI was calculated as weight in kilograms divided by height expressed in meters square. Blood pressure was measured in a sitting position for the right arm to the nearest mm of Hg using mercury sphygmomanometer (Diamond Deluxe; Pune, India). Two readings were taken with a 5 minute interval and the mean of the two was expressed as blood pressure.

### Biochemical parameters

Plasma glucose by glucose oxidase-peroxidase method, serum cholesterol by cholesterol oxidase-peroxidase-4-aminophenazone method, serum triglycerides by glycerol phosphate oxidase peroxidase-4-aminophenazone method and HDL cholesterol via direct method (with polyethylene glycol-pretreated enzymes) were measured using the Hitachi-912 Autoanalyzer (Hitachi, Manheim, Germany). The intra- and inter-assay coefficient of variation (%CV) for these biochemical tests ranged between 3.1% and 7.6% respectively. LDL cholesterol values were derived using Friedewald formula. Insulin concentrations were estimated by enzyme-linked immunosorbent assay (Dako, Glostrup, Denmark). The intra- and inter-assay %CV for insulin measurements were 5.7% and 8.9% respectively. HbA1_c _was measured by high-pressure liquid chromatography using the variant machine (BioRad, Hercules, California, USA). The intra- and inter-assay %CV of HbA1_c _measurements was less than 10%. Insulin resistance was estimated using homeostasis assessment model of insulin resistance (HOMA-IR) calculated as fasting insulin (μU/ml) X fasting glucose (mmol/l) divided by 22.5.

### Quantification of circulating progenitor cells via Flow Cytometry

Peripheral blood progenitor cells were analyzed for surface expression of CD34 and CD133 through direct two-color flow cytometry. Peripheral blood was drawn each at fasting and 2 hr post-load conditions in EDTA vacutainers (BD Biosciences, USA). Prior to staining, samples were blocked with 5% fetal bovine serum and FcR reagent for 20 min. Samples were stained with CD133-PE and CD34-FITC antibodies (Miltenyi Biotech, Germany). Corresponding isotype control IgG1-PE and IgG2a-FITC (Miltenyi Biotech, Germany) antibodies were used. Following red cell lysis, a minimum of 5 lakh events were acquired and scored as per EUROSTAR guidelines [18] using FACS Calibur Flow cytometer (Becton Dickinson, USA). Data were processed using Flowjo software program (Version 7.6.1, Tree Star Inc., USA). The instrument was daily optimized by analyzing the expression of peripheral blood leukocytes with anti-CD4 and anti-CD3 antibodies. The operator who acquired the FACS data was masked to the clinical status of the subjects. Following appropriate gating, CD34^+^, CD133^+ ^and CD133^+^CD34^+ ^cells were enumerated from the lympho-monocyte fraction and cell numbers are represented as number of cells per million cytometric events.

### Statistical Analysis

Cell numbers are expressed as mean ± s.e.m per 10^6 ^events. Normal distribution of the data was confirmed through Kolmogorov-Smirnov test. Skewed distributions were log transformed for statistical analysis. Comparisons between two groups and within groups were performed by the unpaired and paired Student's *t *test respectively for continuous variables. For non-parametric variables, Mann-Whitney's test and Wilcoxon matched pair test were employed. Correlation between cell counts and biochemical parameters were assessed with Spearman's Rank coefficient (ρ). Statistical significance was accepted if the null hypothesis could be rejected at p < 0.05. All analyses were done using Windows - based SPSS statistical software (version15.0).

## Results

### Characteristics of the study population

The morphometric, clinical and metabolic features of the study group are summarized in Table 1. None of the subjects were on medication at the time of recruitment and all provided written consent for the study. The pre-diabetes group had significantly higher body weight (p = 0.026), higher BMI (p = 0.005) and increased waist circumference (p = 0.032). The WHR was also higher for the pre-diabetes group though the increase did not reach level of significance. Among the glucose and insulin profiles, the pre-diabetes group had higher levels of fasting glucose, fasting insulin, post load glucose and post load insulin. Also the pre-diabetes group had greater insulin resistance as measured by HOMA-IR (p = 0.0001). The HbA_1c _levels were significantly higher in pre-diabetes group compared to the NGT group. There was a marginal increase in the neutrophil counts which was approaching significance while the lymphocytes were decreased in the pre-diabetes group (Table 2 p = 0.063). For the benefit of the readers the clinical profiles of IFG and IGT subjects within the pre-diabetes group are further listed in Table 3. As seen in Table 3 the weight of IFG subjects was significantly higher than NGT. Both the IFG and IGT subjects were insulin resistant as measured via HOMA-IR and their glucose and insulin profiles were accordingly higher than the NGT group.

**Table 1:** Anthropometric, clinical and biochemical characteristics of study subjects

Parameters	NGT	Pre-diabetes	p value
	(n = 24)	(n = 26)	
**Anthropometric measures**			

Age (years)	36 ± 2	39 ± 2	0.232

Weight (Kg)	69 ± 2	77 ± 3*	0.026

Body Mass Index (kg/m^2^)	24.0 ± 0.6	26.5 ± 0.6*	0.005

Waist Circumference (cm)	90.3 ± 1.7	98.3 ± 3.1*	0.032

Waist - Hip Ratio	0.96 ± 0.01	0.98 ± 0.01	0.070

Systolic Blood Pressure (mmHg)	119.0 ± 1.8	122.8 ± 2.6	0.240

Diastolic Blood Pressure (mmHg)	76.3 ± 1.6	76.6 ± 1.9	0.992

Biochemical parameters			

**OGTT**			

Fasting plasma glucose (mmol/l)	5.0 ± 0.1	5.7 ± 0.1**	0.0001

2Hr Post plasma glucose (mmol/l)	5.6 ± 0.2	8.1 ± 0.3**	0.0001

Glycated Hemoglobin [HbA1c] (%)	5.4 ± 0.1	5.8 ± 0.1*	0.0013

Fasting Insulin (pmol/l)	49.8 ± 5	95.1 ± 10**	0.0002

2Hr post Insulin (pmol/l)	325.8 ± 49	780.0 ± 75**	0.0001

HOMA-IR	1.9 ± 0.2	4.0 ± 0.4**	0.0001

**Lipid profile**			

Total Cholesterol (mmol/l)	4.6 ± 0.2	4.9 ± 0.2	0.376

Serum Triglycerides (mmol/l)	1.51 ± 0.2	1.9 ± 0.2	0.166

HDL Cholesterol (mmol/l)	1.0 ± 0.04	0.9 ± 0.04	0.459

LDL Cholesterol (mmol/l)	2.9 ± 0.1	3.0 ± 0.2	0.632

VLDL Cholesterol (mmol/l)	0.7 ± 0.1	0.9 ± 0.1	0.163

**Others**			

Serum Creatinine (μmol/l)	79.6 ± 1.8	106.9 ± 27.1	0.364

Hs-CRP (mg/l)	2.5 ± 0.5	3.1 ± 0.5	0.341

Microalbuminuria (μg/mg)	5.2 ± 0.8	7.6 ± 1.5	0.344

Values are expressed as mean ± SEM. Hs - CRP indicates High sensitive C - reactive protein. Glycated Hemoglobin values are expressed in %.*P < 0.05 and **P < 0.001.

**Table 2:** Hematology measurements of study subjects

Parameters	NGT	Pre-diabetes	p value
	(n = 24)	(n = 26)	
WBC count (10^9 ^/l)	6.8 ± 0.2	7.0 ± 0.3	0.717

Neutrophils (%)	51.5 ± 1.4	54.8 ± 1.6	0.076

Lymphocytes (%)	35.5 ± 1.3	32.3 ± 1.1	0.063

Monocytes (%)	8.1 ± 0.3	7.6 ± 0.3	0.148

Eosinophils (%)	4.7 ± 0.8	5.1 ± 0.7	0.727

Basophils (%)	0.18 ± 0.02	0.25 ± 0.04	0.177

RBC count (10^12 ^/l)	5.1 ± 0.1	5.2 ± 0.1	0.750

Platelets (10^9 ^/l)	243.5 ± 7.7	247.8 ± 10.7	0.747

Values are expressed as mean ± SEM. Differential cell count values are expressed in %.

**Table 3:** Anthropometric, clinical and biochemical characteristics of study subjects

Parameters	NGT	IFG	IGT
	(n = 24)	(n = 09)	(n = 17)
**Anthropometric measures**			

Age (years)	36 ± 2	38 ± 2	40 ± 2

Weight (Kg)	69 ± 2	82 ± 4**	74 ± 3

Body Mass Index (kg/m^2^)	24.0 ± 0.6	27.0 ± 1.3*	26.2 ± 0.7*

Waist Circumference (cm)	90.3 ± 1.7	97.0 ± 2.2*	99.0 ± 4.7

Waist - Hip Ratio	0.96 ± 0.01	0.97 ± 0.01	0.98 ± 0.01

Systolic Blood Pressure (mmHg)	119.0 ± 1.8	120.0 ± 1.9	124.3 ± 3.8

Diastolic Blood Pressure (mmHg)	76.3 ± 1.6	75.6 ± 1.9	77.1 ± 2.7

**OGTT**			

Fasting plasma glucose (mmol/l)	5.0 ± 0.1	5.7 ± 0.1***	5.7 ± 0.2***

2Hr Post plasma glucose (mmol/l)	5.6 ± 0.2	6.8 ± 0.3**	8.8 ± 0.2*** ^##^

Glycated Hemoglobin [HbA1c] (%)	5.4 ± 0.1	5.6 ± 0.1	5.9 ± 0.1***

Fasting Insulin (pmol/l)	49.8 ± 5	93.7 ± 19 **	95.8 ± 11***

2Hr post Insulin (pmol/l)	325.8 ± 49	653.1 ± 121**	1259 ± 231***

HOMA-IR	1.9 ± 0.2	3.9 ± 0.8***	4.0 ± 0.5***

**Lipid profile**			

Total Cholesterol (mmol/l)	4.6 ± 0.2	4.8 ± 0.4	4.9 ± 0.2

Serum Triglycerides (mmol/l)	1.51 ± 0.2	2.1 ± 0.4	1.8 ± 0.2

HDL Cholesterol (mmol/l)	1.0 ± 0.04	1.0 ± 0.09	1.0 ± 0.03

LDL Cholesterol (mmol/l)	2.9 ± 0.1	2.9 ± 0.3	3.1 ± 0.2

VLDL Cholesterol (mmol/l)	0.7 ± 0.1	0.9 ± 0.2	0.8 ± 0.1

**Others**			

Serum Creatinine (μmol/l)	76.6 ± 2.3	73.7 ± 2.6	124.5 ± 41.2**^#^**

Hs-CRP (mg/l)	2.5 ± 0.5	2.0 ± 0.2	3.7 ± 0.6

Microalbuminuria (μg/mg)	5.2 ± 0.8	4.2 ± 0.6	9.4 ± 2.1

Values are expressed as mean ± SEM. Hs - CRP indicates High sensitive C - reactive protein. Glycated Hemoglobin values are expressed in %. *P < 0.05, **P < 0.001 &*** *P *< 0.0001 vs NGT and # *P *< 0.05 & ## *P *< 0.0001 vs IFG.

### Circulating progenitor cells in NGT versus Pre-diabetes group

**Figure 1:**
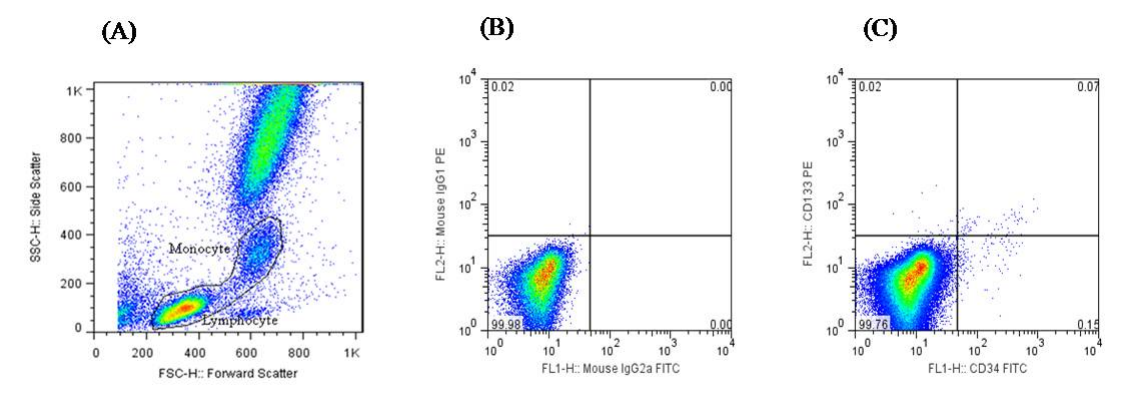
**Representative FACS dot plot**. Forward and side scatter of the peripheral blood samples depicting lymphocyte and monocyte fraction for gating (A). Quadrant settings of the isotype control (B) and test sample (C) stained with their respective antibodies.

Upon morphological gating of the lympho-monocyte fraction based on forward and side scatter, the CD34^+^, CD133^+ ^and CD133^+^CD34^+ ^cells were sorted following gating with appropriate fluorophore labeled corresponding isotype control antibodies (Figure 1). No differences were observed in the fasting levels of CD34^+ ^cells between NGT and pre-diabetes subjects (1401 ± 417 vs 1751 ± 483, p = 0.589) or for CD133^+ ^cells (34 ± 12 vs 52 ± 22, p = 0.874). Here the cell numbers are depicted for every one million cytometric events counted. Similarly no differences were seen in the circulation for fasting levels of CD133^+^CD34^+ ^cells between the two groups (347 ± 114 vs 295 ± 69, p = 0.392 for NGT versus pre-diabetes respectively).

**Figure 2:**
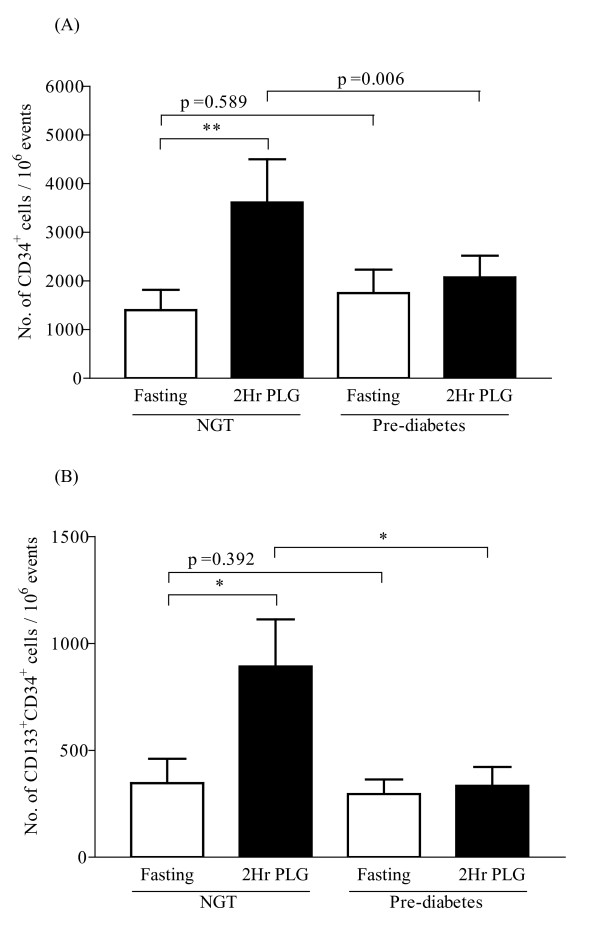
**Circulating progenitor cells in study subjects following glucose challenge**. CD34^+ ^cells (A) and CD133^+^CD34^+ ^cells (B) represented per million cytometric events at fasting and 2 hour post glucose challenge (2Hr PLG). Bar graphs summarize data as mean ± SEM; * p < 0.05 and ** p < 0.01 represents statistical significance.

However at 2 hour post glucose load there was almost a 3-fold increase in the CD34^+ ^cell count in the NGT group (p = 0.003) which appeared to be attenuated in the pre-diabetes group with the p value approaching significance (refer to Figure 2A, p = 0.066). No increase in the CD133^+ ^cells following glucose load was seen in either of the study groups (data not shown). Similarly a 2.5-fold increase was seen for CD133^+^CD34^+ ^cells in the NGT group (p = 0.019) at 2 hours following 75 g glucose load, which was blocked in pre-diabetes group (Figure 2B, p = 0.024).

**Figure 3:**
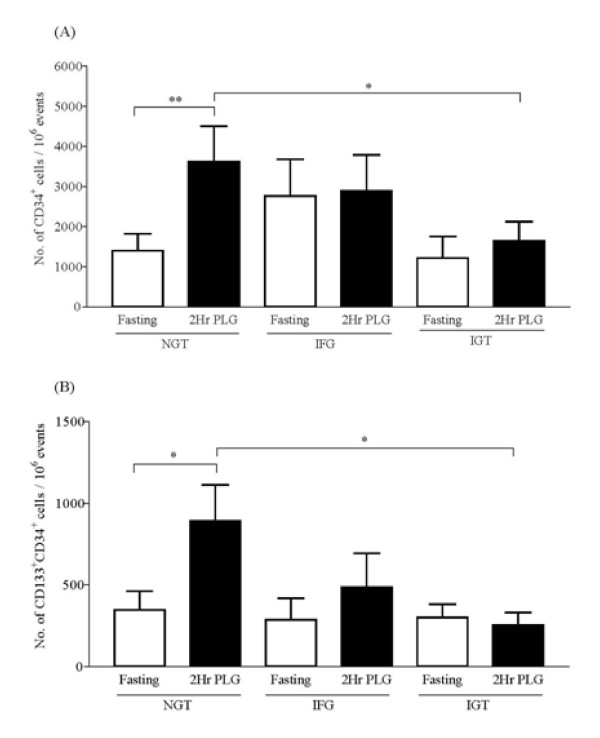
**Circulating progenitor cells in NGT, IFG and IGT subjects following glucose challenge**. CD34^+ ^cells (A) and CD133^+^CD34^+ ^cells (B) represented per million cytometric events at fasting and 2 hour post glucose challenge (2Hr PLG). Bar graphs summarize data as mean ± SEM; * p < 0.05 and ** p < 0.01 represents statistical significance.

Furthermore when the pre-diabetes group was further split into IFG and IGT, the glucose mediated increase in CD34^+ ^and CD133^+^CD34^+ ^was significantly blocked for the IGT subjects (Figure 3). Intriguingly for the IFG group there appeared to be an increase in the fasting levels of CD34^+ ^cells compared to NGT (Figure 3A) but the p value did not reach significance (p = 0.133). With regard to CD133^+^CD34^+ ^cells the fasting levels of these progenitor cells did not vary among the group though the glucose-load induced increase appeared blunted for IFG (Figure 3B). It should however be noted that these observations are made with limited number of volunteers subjected to a restricted OGTT analysis which scores cell numbers only at fasting and 2 hour post load stages. This hence warrants for a detailed analysis of progenitor cell kinetics every 30 minutes following glucose challenge in future studies.

### Correlation Analysis

Spearman's Rank correlation analysis was performed to determine correlation between progenitor cell numbers and biochemical parameters. A positive correlation was observed between CD34^+ ^and CD133^+^CD34^+ ^cells both during fasting and 2 hour post glucose load. The Rho value for fasting cell counts was 0.270 (p = 0.058) while for 2 hour values the Rho between the two cell types was 0.361 (p = 0.01). When fasting cell counts for CD34^+ ^were analyzed, no appreciable correlation was seen with fasting profiles of glucose or insulin, however fasting CD34^+ ^cells showed a near significant inverse correlation with cholesterol (ρ = -0.263, p = 0.064), LDL (ρ = -0.259, p = 0.070) and serum creatinine (ρ = -0.249, p = 0.084). The fasting cell counts for CD133^+^CD34^+ ^cells exhibit a near significant and significant positive correlation with fasting glucose (ρ = 0.245, p = 0.086) and post load glucose (ρ = 0.297, p = 0.036) respectively. Serum creatinine levels exhibited inverse correlation with post glucose load cell counts of CD133^+^CD34^+ ^cells (ρ = -0.312, p = 0.023). Intriguingly the CD34^+ ^cell counts following glucose load demonstrated inverse correlation with neutrophils, with ρ = - 0.330 (p = 0.019).

## Discussion

In the current study, we report a 2 to 3-fold increase in the circulating levels of CD34^+ ^and CD133^+^CD34^+ ^progenitor cells in healthy Asian Indian males in response to 75 g glucose load. This increase in progenitor cell counts is however attenuated in pre-diabetes subjects particularly IGT. To the best of our knowledge this is the first study to identify an increase in circulating progenitor cells in response to glucose load in healthy individuals and its attenuation in IGT. A positive correlation was seen between CD34^+ ^and CD133^+^CD34^+ ^cells both at fasting and post load stages, confirming a previous finding that double positive cells are a subset of the CD34^+ ^pool [19]. As previously reported we also observed an inverse correlation of fasting CD34^+ ^cell counts with cholesterol and LDL [19], however the p values for these correlations did not reach significance possibly due to small sample size. Intriguingly the CD34^+ ^cell counts following glucose load showed inverse correlation with neutrophils (p = 0.019). Additionally the post load CD133^+^CD34^+ ^counts were inversely correlated with serum creatinine.

Progenitor cells besides giving rise to EPCs can integrate into skeletal muscle, neurons or myo-endothelial cells [1]. They exhibit better dispersion in host muscle tissue following cryo injury and can form capillary like structures [20]. Similarly CD133^+^CD34^+ ^cells exhibit a strong correlation with degree of engraftment in multiple myeloma patients undergoing autologous transplantation [21]. It is of interest that a recent study also claims that HSC transplantation imparts insulin independency in early type I diabetes patients [22]. Although the knowledge about mobilization of various fractions of haematopoietic stem cells and the endogenous processes governing physiological organ recoveries mediated by them are scarce, it is tempting to speculate that increases in circulating progenitor cell counts in response to glucose load is possibly the body's precautionary response to protect various tissues and organs from glycemic insults. In the likelihood of this possibility the inverse correlation of CD133^+^CD34^+ ^cells (ρ = -0.312, p = 0.023) with serum creatinine is indeed interesting. However these findings need to be tested and characterized in experimental models of diabetes.

Unlike a recently published study by Fadini et.al. [14], we did not observe any variations in the fasting cell counts of CD34^+ ^progenitor cells between the two groups possibly due to small sample size. We however observed an attenuated response at 2 hours post glucose challenge for CD34^+ ^and CD133^+^CD34^+ ^cells in the pre-diabetes group compared to the NGT group. Although the mechanisms behind this blunted response and the functional consequences of this aberration are presently unknown it can be envisaged that hostile vascular environment in pre-diabetes either due to reactive oxygen species or inflammation may affect the mobilization of progenitor cells from bone marrow. It should be noted that pre-diabetes is associated with increased oxidative stress and circulatory pro-inflammatory cytokines [15,23,24]. Given that the post glucose load-cell counts for CD34^+ ^inversely correlated with neutophil counts, it is likely that decrease in progenitor count is an effect of increased inflammatory state. Since we did not assess the oxidative stress in these subjects, it is difficult at this stage to comment on the influence of reactive oxygen species on mobilization of progenitors from bone marrow in pre-diabetes subjects. Alternatively it is likely that reduction in post load progenitor cell counts in pre-diabetes subjects is due to accelerated progenitor cell senescence which unfortunately was not investigated in this study. Ageing is known to restrict the bone marrow reserve of progenitor cells and reduce circulating numbers of these cells [25,26]. Hence this study was performed with age matched younger subjects. The other advantage of this study is the recruitment of drug naïve NGT and pre-diabetes subjects. Thus any compounding effects of either medication or age are minimized and the study provides a reasonable indication of influence of glucose challenge on progenitor cell count. It is likely that blunted post load response of progenitor cells may precede the actual decrease seen under fasting conditions for these cells during the course of developing diabetes [7,10], thereby suggesting that insults to vasculature and possibly other organs occur prior to the onset of overt diabetes diagnosed through fasting glucose levels [27]. Finally whether similar progenitor dynamics with regard to post glucose challenge is seen in other populations prior to or during the onset of diabetes is currently unknown and this points to need for replication of the findings in other populations.

### Limitations

There are certain limitations of the current study. For example due to small sample size certain observations did not reach levels of statistical significance. Similarly although in the IFG subjects there appears to be an increase in the fasting levels of CD34^+ ^cells as opposed to NGT, it fails to reach statistical significance. Additionally due to small sample size we could not perform sub-group analysis and being observational in nature, the causative factors could not be determined. The other limitation of the study is a restricted OGTT analysis with measurement of glucose and insulin only at fasting and 2 hour post glucose challenge. A detailed analysis every 30 minutes would give a better perspective on progenitor cell kinetics as a function of metabolic parameters. Finally the study addresses the progenitor cell dynamics in Asian Indian male subjects, whether similar dynamics are seen in female subjects' remains to be seen.

## Conclusions

Glucose challenge increases circulating levels of CD34^+ ^and CD133^+^CD34^+ ^haematopoietic stem cells in healthy subjects which is compromised in subjects with IGT.

## Abbreviations

OGTT: Oral Glucose Tolerance Test; IFG: Impaired fasting glucose; IGT: Impaired glucose tolerance; NGT: Normal glucose tolerance; HOMA-IR: Homeostasis model assessment of insulin resistance; EPC: Endothelial progenitor cell; HSC: Haematopoietic stem cell; CAD: Coronary artery disease; CVD: Cardiovascular disease; FACS: Fluorescence assisted cell sorting; BMI: Body mass index; WHR: Waist to hip ratio.

## Competing interests

The authors declare that they have no competing interests.

## Authors' contributions

AAN executed the study and analyzed data, VM screened patients and reviewed manuscript, SSB analyzed data and reviewed manuscript, SB analyzed data and MD planned the study, reviewed the data and wrote the manuscript. All authors have read and approved the final manuscript.

## Pre-publication history

The pre-publication history for this paper can be accessed here:

http://www.biomedcentral.com/1472-6823/11/2/prepub
